# The Susceptibles, Chancers, Pragmatists, and Fair Players: An Examination of the Sport Drug Control Model for Adolescent Athletes, Cluster Effects, and Norm Values Among Adolescent Athletes

**DOI:** 10.3389/fpsyg.2020.01564

**Published:** 2020-07-10

**Authors:** Adam R. Nicholls, Andrew R. Levy, Rudi Meir, Colin Sanctuary, Leigh Jones, Timothy Baghurst, Mark A. Thompson, John L. Perry

**Affiliations:** ^1^Department of Sport, Health and Exercise Science, University of Hull, Hull, United Kingdom; ^2^Department of Psychology, Edge Hill University, Ormskirk, United Kingdom; ^3^School of Health and Human Sciences, Southern Cross University, Lismore, NSW, Australia; ^4^School of Environmental and Life Sciences, The University of Newcastle, Callaghan, NSW, Australia; ^5^Hong Kong Rugby Foot-Ball Union, Hong Kong, Hong Kong; ^6^College of Education, Florida State University, Tallahassee, FL, United States; ^7^Department of Psychology, Mary Immaculate College, Limerick, Ireland

**Keywords:** adolescence, attitudes, doping, performance-enhancing drugs, norm values

## Abstract

Although there are few high-profile cases of adolescent athletes being caught doping, up to a third of young athletes may dope. In order to generate a more accurate understanding of why adolescent athletes dope, it is important to validate models that help to explain this behavior. The aims of this study were 3-fold: firstly, to test the Sport Drug Control Model for Adolescent Athletes (SDCM-AA); secondly, to generate athlete profiles that would help quantify the proportion of athletes who are at risk of doping; and thirdly, to create norm values for the Adolescent Sport Doping Inventory (ASDI), which would allow national doping organizations, sporting organizations, and clubs to benchmark the scores of their athletes for key psycho-social variables linked to doping. A total of 2208 adolescent athletes from the United Kingdom, Australia, Hong Kong, and the United States completed the ASDI. The data presented an appropriate fit to the SDCM-AA model, in which 54% of the variance in susceptibility to doping was explained in the model, and 44.8% of attitudes toward doping was accounted for. Four distinct clusters of athletes emerged: the Susceptibles (i.e., identified with the benefits of doping, were willing to cheat, and viewed little threat), the Chancers (i.e., identified with the benefits of doping, scored high on willingness to cheat, and were highly influenced by their reference group, but had an average score for threat, self-esteem, and legitimacy), the Pragmatists (i.e., did not engage with any aspects of doping, but were more susceptible than the fair players), and Fair Players (i.e., high levels of sportspersonship, unwilling to cheat, and viewed doping as a threat). The revised SDCM-AA appears a valid model that helps explain the factors associated with doping attitudes and doping susceptibility. Adolescent athletes can be classified into one of four clusters, in relation to doping. Their cluster group could influence the content of the anti-doping education they receive.

Doping refers to taking performance-enhancing drugs (PEDs) or using banned methods among sports, as identified on the World Anti-Doping Agency (World Anti-Doping Agency [WADA], 2018), and represents cheating in sport (Kavussanu, 2019). Adolescence refers to the period in which a person is aged between 12 and 18 years of age (Weiss and Bredemeier, 1983). This is the period in a person’s life when attitudes and values are formed and then take shape (Cieciuch et al., 2015; Döring et al., 2015; Kjellström et al., 2017), which is important to note, as attitudes are thought to be a key factor in influencing whether athletes will dope or not (see Ntoumanis et al., 2014; Nicholls et al., 2017a; for reviews). Although there are few high-profile cases of children or adolescents being found guilty of doping, up to 30% of adolescents may dope (i.e., Gradidge et al., 2010). In the Gradidge study, adolescent athletes reported anti-doping rule violations, which included using growth hormones, anabolic androgenic steroids (AAS), and ephedrine. The figures reported by Gradidge et al. are somewhat higher than those in the European School Survey Project on Alcohol and other Drugs report (ESPAD, 2015). In the ESPAD report, 96,043 young people from 35 European countries were surveyed. Of these, around 1% of school pupils took AAS, and the abuse of AAS varied across different countries, and was as high as 4% in Bulgaria among males and females. In Bulgaria, 7% of young males abused AAS, whereas a 5% of Cypriot young males used AAS. It should be noted that some of the participants in ESPAD (2015) may have been gymgoers rather than athletes, who took AAS for enhanced physical appearance such as added muscle or reduced body fat, rather than to aid sporting performance (Klimek and Hildebrandt, 2018). Furthermore, the ESPAD survey did not measure other banned substances or methods that were reported in the Gradidge et al. study, such as growth hormones, ephedrine, or blood doping. Therefore, doping may be higher than the figure reported by ESPAD. Not only do banned substances represent a physical threat to athletes who dope (Bird et al., 2016), doping is also associated with an increased risk of committing suicide (Lindqvist et al., 2014). It is important that scholars understand more about the antecedents of doping or factors associated with doping among young athletes (Nicholls et al., 2020). This knowledge and understanding would have the potential to reduce the prevalence of these behaviors among this group of athletes.

At the present time, however, only three models have attempted to explain why young athletes dope. These are the Social-Cognitive Model (Zelli et al., 2010), the Integrated Model of Doping Behavior (IMDB; Lazuras et al., 2015), and the Sport Drug Control Model for Adolescent Athletes (SDCM-AA; Nicholls et al., 2015).

## Social-Cognitive Model

Ten different high schools from Italy participated in testing this model, which involved two assessed periods 4–5 months apart. A total of 864 adolescent athletes completed both assessments. This model predicts that a number of factors (e.g., doping attitude, subjective norms, perceived behavioral control, doping self-regulatory efficacy, and doping moral disengagement) contribute to form an athlete’s intention to dope, which, in turn, predicts doping behavior over time. Zelli et al. (2010) found support for this model, as intentions to dope at Time 1 predicted doping use 4–5 months later. A possible limitation of this model is that it was tested exclusively with Italian athletes, so little is known about the generalizability of the model to athletes from other countries. Further, it does not include other constructs that appear important in shaping doping attitudes, such as the perceived legitimacy of anti-doping organizations, personal morality, and perceptions of deterrents, which feature in other models (e.g., Donovan et al., 2002; Nicholls et al., 2015).

## The Integrated Model of Doping Behavior

With a sample of adolescent athletes from northern Greece, Lazuras et al. (2015) expanded the integrated model previously developed by Barkoukis et al. (2013), by including demographic variables such as age and gender as distal variables. The IMDB (Lazuras et al., 2015) includes distal (e.g., achievement goals, motivational regulations, and moral orientations) and proximal predictors of doping intentions (e.g., outcome expectancy beliefs, social norms, and self-efficacy beliefs). Regression analyses revealed that the model predicted 57.2% of the variance in intentions to dope. Furthermore, doping attitudes, social norms, and self-efficacy beliefs added 34.4% of the variance in intentions, on top of distal predictors. A potential limitation of the integrated model is that the motivational variables were included as distal predictors, rather than proximal predictors, because motivational variables may have a direct effect on doping intentions (Ntoumanis et al., 2014). Additionally, the integrated model does not include other factors that appear important in relation to doping, such as threat appraisals, benefit appraisals, views on the legitimacy of anti-doping organizations, and personality. These constructs all appear in other models, such as the Sport Drug Control Model (SDCM; Donovan et al., 2002) and the SDCM-AA (Nicholls et al., 2015).

## The Sport Drug Control Model for Adolescent Athletes

The SDCM-AA (Nicholls et al., 2015) was adapted specifically for adolescent athletes from the SDCM (Donovan et al., 2002). Nicholls et al. interviewed 11 coaches from four countries regarding the applicability of the original SDCM (Donovan et al., 2002) to adolescent athletes and found support for the applicability of the SDCM to adolescent athletes, with some minor alterations, which are described after presenting the SDCM.

The SDCM (Donovan et al., 2002) integrates three behavioral science frameworks (i.e., threat/fear appeals, social cognition, and instrumental and normative approaches). Donovan et al. (2002) proposed that intentions/attitudes toward doping were the key factor that influenced whether an athlete would dope or not. Donovan et al. (2002) proposed that doping attitudes are influenced by six different constructs (i.e., threat appraisals, benefit appraisals, reference group opinions, morality, legitimacy, and personality). Threat relates to negative health consequences of doping and also the likelihood of being caught. Benefit appraisals include the gains that can potentially occur from doping, such as increased earnings, fame, or winning competitions. Reference group opinion relates to the extent that parents, coaches, friends, or spouses approve or disapprove of doping, and the influence they can exert upon athletes. Morality relates to whether athletes believe doping is right or wrong, while legitimacy is about how athletes perceive organizations that police doping. Finally, personality was also believed to influence attitudes toward doping. Two studies have quantitatively examined the SDCM (Gucciardi et al., 2011; Jalleh et al., 2014). With a sample of 670 elite athletes from Australia, Gucciardi et al. (2011) reported that morality (cheating), threat appraisals, and benefit appraisals were strongly associated with doping attitudes. Self-esteem, legitimacy, and reference group opinion, however, were not associated with doping attitudes.

Utilizing another sample of elite athletes, Jalleh et al. (2014) found that morality, reference group opinion, and legitimacy were associated with doping attitudes. Although these two studies provide support for the SDCM, it should be noted that both studies tested the constructs of the SDCM exclusively with elite athletes from Australia only. Rad et al. (2018) argued that results of studies with participants from one country might not be applicable to other countries. There is evidence that there may be differences in participants from different countries in relation to key elements of the SDCM. These include appraisal (e.g., Imada and Ellsworth, 2011), morality (e.g., An and Trafimow, 2014), social norms (e.g., Shen et al., 2011), self-esteem (Brown and Cai, 2010), and personality (Kövi et al., 2019). For these reasons, it could be argued that doping models could be tested among athletes residing in different countries. Another potential issue of applying the SDCM to adolescent athletes from different countries is that the SDCM was designed and tested among adult athletes. Scholars such as Compas et al. (2001) suggested that adolescents should not be treated as mini-adults and that theoretical models should be designed for the specific population. This is particularly applicable to models that include attitudes due to the development and formation of this construct. It is accepted that adolescents’ attitudes have not fully formed during this part of their life, as they typically develop and take shape during adolescence (Cieciuch et al., 2015; Döring et al., 2015; Kjellström et al., 2017). As such, it appears imperative to not generalize attitudes of adult athletes to those of adolescent athletes. Although the SDCM was not designed to predict doping specifically among adolescents, the central construct of this model, attitudes/intentions predict doping behavior among adolescent athletes. Two studies revealed that intentions to dope predicted actual doping behavior. Featuring a sample of 1022 athletes, Zelli et al. (2010) assessed intentions at Time 1 (along with other constructs) and doping behavior at Time 2, 4–5 months later, with a sample of adolescent athletes. They also found that intentions predicted doping behavior in a prospective study (Lucidi et al., 2013) in which doping behavior and a variety of constructs were examined across two time points among 1975 adolescent athletes. Intentions at Time 1 predicted actual doping behavior at Time 2. Additionally, Ntoumanis et al. (2014) meta-analysis, which contained samples of adolescents, found that doping attitudes predicted doping behavior.

For the aforementioned reasons, Nicholls et al. (2015) re-examined the SDCM (Donovan et al., 2002), in order to assess its accuracy with adolescent athletes. Overall, Nicholls et al. (2015) found support for the original SDCM. Support was found for the influence of threat appraisals, benefit appraisals, reference group opinions, morality, legitimacy, and personality on attitudes toward doping. The coaches also identified additional factors they thought were specifically relevant to adolescent athletes in the development of attitudes toward doping. These included participation level, stress, age or maturation, ethnicity, and country of residence. In particular, some of the coaches interviewed in Nicholls et al. (2015) had worked in different countries and believed there were differences in attitudes toward doping among athletes from different countries. That is, in some countries, there are much more favorable attitudes toward doping among young athletes, in comparison to athletes from other countries. In regards to stress, the coaches argued that high expectations on athletes, which causes them to worry, may lead them to make poor decisions and take PEDs. Another coach argued that it was the physical toll of playing competitive sport at young age, particularly toward the end of the season, that could lead to some athletes developing a favorable attitude toward doping.

Doping susceptibility was not included in the SDCM (Donovan et al., 2002) as a factor that predicted doping behavior. This construct was, however, included in the SDCM-AA (Nicholls et al., 2015). Doping susceptibility is “the absence of a firm resolve not to engage in doping activities or to give any consideration at all to an offer to do so” (Gucciardi et al., 2010, p. 481). The coaches in the Nicholls et al. (2015) study believed that doping susceptibility was an important construct, which was linked to doping attitudes and would influence whether or not adolescent athletes would dope, so was included in the SDCM-AA. In support of this addition, both Barkoukis et al. (2014) and Blank et al. (2016) reported that doping susceptibility was a proxy for doping behaviors, when it is associated with positive attitudes toward doping. To date, however, researchers have assumed a concomitant relationship between doping susceptibility and doping behavior, without assessing this directly. Nevertheless, susceptibility appears to be a predictor of substance use among non-athletic adolescents. For example, several studies have longitudinally assessed the relationship between susceptibility and both smoking (e.g., Jackson, 1998) and alcohol use (Andrews et al., 2008; Cranford et al., 2010) among adolescents. These studies that susceptibility was associated with a greater prevalence of smoking and alcohol use. Further, reducing susceptibility appears to lower alcohol for up to 1 ^1^/_2_ years later among adolescents (Jackson et al., 2016), illustrating the possible importance of susceptibility among adolescents in regard to a doping context.

The constructs of the SDCM-AA were used to develop the Adolescent Sport Doping Inventory (ASDI; Nicholls et al., 2019a). However, the SDCM-AA, which includes influence of threat appraisals, benefit appraisals, reference group opinions, morality, legitimacy, self-esteem, participation level, stress, age or maturation, ethnicity, and country of residence as factors that predict attitudes toward doping and doping susceptibility, has not been quantitatively examined to assess its validity.

## Clusters and Psycho-Social Variables Associated With Doping

Another potential use of the SDCM-AA (Nicholls et al., 2015) and the ASDI (Nicholls et al., 2019a) is to identify key psycho-social factors associated with doping among adolescent athletes, which can then be used to formulate cluster scores or profiles for each athlete. Although cluster analyses have not been extensively used in the doping literature, they have been used in other domains such as risk behaviors (Meader et al., 2016), attitudes toward science (Sheldrake et al., 2017), and enhancing clinical practice (Windgassen et al., 2018), and may be of benefit to researchers in the field of doping. Clustering may be of interest to doping scholars and national anti-doping organizations because it facilitates the quantification of the proportion of athletes who may be at high risk of taking PEDs, along with those who are a medium risk of doping, and athletes who are a low risk of doping. Researchers could also assess whether and how these proportions change over time, which would offer new knowledge within the field of anti-doping (Sheldrake et al., 2017).

Additionally, understanding more about how psycho-social factors associated with doping co-occur can be useful in developing prevention strategies (Meader et al., 2016). For these reasons, clustering may be a useful addition to the doping literature, which has implications for the development and monitoring of anti-doping education. Despite the potential benefits of clustering, there are few examples in the doping literature. One exception is the study by Barkoukis et al. (2011), who examined doping behavior in response to clusters of motivation, achievement goals, and sportspersonship. Amotivated athletes, whose behavior has a lack of intentionality (Vallerand, 2001), scored higher on past doping use and intentions to dope than intrinsically (i.e., behavior driven by satisfaction) or extrinsically (i.e., behavior driven by external rewards) motivated athletes. Mastery Orientated (i.e., participating in sport for self-improvement) athletes were less likely to have doped than athletes who were Approach Orientated (i.e., participating in sport to demonstrate superiority over others). There were no significant differences in past doping use among the clusters of high and low levels of sportspersonship. Although not cluster analyses *per se*, Duncan et al. (2018) interviewed 21 young adults and developed four specific profiles that reflected beliefs, perceptions, motives, and circumstances associated with athletes considering doping. This research detailed how some young athletes may experience a breaking point, which could result in them taking PEDs. Therefore, identifying clusters or athlete profiles could be useful to sporting organizations, national anti-doping organizations (NADOs), or education authorities in identifying athletes who may be at risk of doping.

## Norm Values

A notable omission from the doping literature, particularly for adolescent athletes, is a set of norm values for scores in the key psycho-social variables associated with doping. Given that athletes as young as 10-year-olds may dope (see Nicholls et al., 2017a) and up to 30% of adolescents dope (Gradidge et al., 2010), this age represents a high-risk period in which some young people may initiate doping (Lazuras et al., 2015). For these reasons, providing national anti-doping organizations, sports governing bodies, and coaches with norm values so that they can benchmark their athletes’ scores will allow organizations and coaches to identify and monitor athletes who are at risk of taking PEDs.

To address the aforementioned limitations, the aim of this study was 3-fold: firstly, to test the SDCM-AA (Nicholls et al., 2015); secondly, to create psycho-social doping cluster scores; and thirdly, to create norm values for adolescent athletes that can be used by a variety of stakeholders interested in doping. The SDCM-AA model has not yet been subject to empirical testing, so formulating specific hypotheses was not deemed appropriate.

## Materials and Methods

### Participants

A total of 2500 questionnaires were distributed to sports organizations, schools, coaches, and sports clubs, with 2208 competitive athletes (male *n* = 1456, female *n* = 751, unspecified *n* = 1) returning their questionnaire. The athletes were aged between 12 and 18 years of age (*M* age = 16.36, *SD* = 1.69). This sample resided in the United Kingdom (*n* = 1, 226), Australia (*n* = 427), United States (*n* = 299), and Hong Kong (*n* = 256). Athletes competed at beginner (*n* = 205), amateur (*n* = 1, 469), semi-professionally for a club (*n* = 200), professionally for a club (*n* = 40), county or state (*n* = 147), national (*n* = 105), or international level (*n* = 34). Eight athletes failed to report their competitive playing level. Of the 2208 athletes that were featured in this study, 2107 were featured across the seven studies in the paper by Nicholls et al. (2019a), so the sample was not analyzed altogether. Kirkman and Chen (2011) provided guidance on submitting multiple submissions from the same dataset. They suggested that it is appropriate when different research questions are addressed and each submission will make a unique contribution to the literature. The study by Nicholls et al. (2019a) was concerned with developing and validating the ASDI, whereas the present study was concerned with testing the SDCM-AA (Nicholls et al., 2015), creating psycho-social doping cluster scores, and generating norm values. As such, the aims of Nicholls et al. (2019a) and the current study are different.

### Measure

#### Adolescent Sport Doping Inventory

The 43-item ASDI (Nicholls et al., 2019a) assessed psycho-social variables that are associated with both attitudes toward doping and doping susceptibility. The ASDI was developed in response to a poor model fit of the Performance Enhancement Attitude Scale (PEAS; Petr czi and Aidman, 2009) among adolescent athletes (Nicholls et al., 2017b) and the need to develop a valid questionnaire to assess psycho-social doping variables among adolescent athletes. The ASDI contains nine subscales: attitudes (e.g., “Legalizing PEDs would benefit my sport”), threat (“I would suffer serious health complications if I took PEDs”), benefit (e.g., “Taking PEDs could help me keep my place in the team or training squad”), self-esteem (e.g., “I am worth being in the team/squads that I am currently play for”), cheating (e.g., “I would cheat if I knew I won’t get caught”), legitimacy (e.g., “Drug tests are very thorough”), reference group opinion (e.g., “What other people think about PEDs influences my decision on whether I would ever take them or not”), stress (e.g., “Competing in sport makes me feel anxious or worried”), and susceptibility (e.g., “I would be tempted to take PEDs, if I knew they would increase my performance”). Attitudes and threat both contain four questions each, whereas the subscales for benefit, esteem, cheating, legitimacy, reference group opinion, stress, and susceptibility all have five questions each. All questions were all answered on a seven-point Likert-type scale, anchored at 1 = “*Strongly Disagree*” and 7 = “*Strongly Agree.*” Nicholls et al. (2019a) reported a good confirmatory factor analysis model fit for the ASDI: χ^2^(824) = 1440.403, CFI = 0.954, TLI = 0.950, SRMR = 0.039, RMSEA = 0.035 (90% CI = 0.032,0.038). Further, Nicholls et al. provided support for the convergent validity of the ASDI, as psycho-social doping variables were associated with situational temptation, honesty and humility, maturation, motivational climate, the coach–athlete relationship, stress, coping, achievement goals, and coach behavior.

### Procedure

Ethical approval was obtained from a university departmental ethics committee. Following this, invitation letters and e-mails were distributed to schools, sports clubs, and governing bodies to recruit athletes for this study. Participants who agreed to participate, completed demographic information and the ASDI (Nicholls et al., 2019a) either online or via pen and paper. All athletes completed the ASDI in English.

### Data Analyses

Before testing the SDCM-AA (Nicholls et al., 2015) model, we first sought to examine the extent to which the ASDI model was invariant across the sample. Specifically, we tested model invariance by gender, country, and skill level using multi-group CFA in MPlus Version 7 (Muthén and Muthén, 1998–2012). We followed the same four-step process for each test of invariance. Firstly, configural invariance was assessed replicating the model across sample groups. Second, metric invariance was assessed by constraining factors. Third, scalar invariance was assessed by constraining factors and intercepts, and fourth, residual invariance was assessed by constraining factors, item intercepts, and factor means. We determined measurement invariance using Cheung and Rensvold (2002) recommendation of ΔCFI ≤ 0.01 at each step.

To test the SDCM-AA (Nicholls et al., 2015), we used the nine ASDI (Nicholls et al., 2019a) subscales and demographic variables in a structural equation model (SEM). The SDCM-AA infers that doping attitudes are determined by threat, benefit, self-esteem, cheating^1^, legitimacy, and reference group. In turn, Nicholls et al. (2015) hypothesized that attitudes predicted susceptibility to doping by a reference group. SEM was carried out using MPlus version 7 (Muthén and Muthén, 1998–2012), with each factor indexed by all of its items from the ASDI with no cross-loadings or correlated error terms. Potential moderating variables of gender, country of residence, and skill level were examined using multi-group SEMs, where all measurement components were constrained, allowing structural paths to be freely estimated within each group.

To further examine determinants of attitudes and susceptibility toward doping, we sought to examine clusters within the data and if these were predictive of doping attitude and susceptibility. To do so, we adopted a two-stage approach utilized by Lucidi et al. (2019), initially conducting a hierarchical cluster analysis (Ward’s method) in SPSS 26.0 using the squared Euclidean distance measure to identify the number of cluster groups based on flattening of the dendrogram. Next, we employed *k*-means, non-hierarchical clustering to detect the best-fitting solution. With clusters identified, we tested a one-way ANOVA with cluster as the grouping variable to determine effects on doping attitude and susceptibility. Planned comparisons were examined between each cluster. To correct for multiple comparisons, we adopted Benjamini and Hochberg (1995) false discovery rate. This method calculates a *q* value by which *p* can be compared to identify false discoveries. A *p* value greater than *q* indicates a non-significant effect. Finally, we established normative values using percentile scores.

## Results

Preliminary screening of data found no missing data from non-demographic responses and no problematic outliers. Omega point estimates were used to assess internal consistency. All scales presented satisfactorily (threat = 0.86, benefit = 0.93, self-esteem = 0.90, cheating = 0.90, legitimacy = 0.90, reference group = 0.92, attitude = 0.85, stress = 0.86, susceptibility = 0.93). Measurement invariance was examined in multi-group CFAs for gender, country, and skill level. Across each model, measurement invariance was supported (ΔCFI < 0.01; Table 1).

**TABLE 1:** Measurement invariance testing for country of residence, gender, and skill level.

**Model**	**χ^2^**	***df***	**Δχ^2^**	**Δ*df***	**CFI**	**ΔCFI**	**TLI**	**SRMR**	**RMSEA (90% CI)**
**Gender**									
Configural invariance	4501.12	1648	–	–	0.934	–	0.928	0.034	0.041 (0.039,0.042)
Metric invariance	4541.23	1682	40.11	34	0.934	0.000	0.929	0.035	0.040 (0.039,0.042)
Scalar invariance	4656.88	1716	115.65	34	0.932	0.002	0.928	0.035	0.041 (0.039,0.042)
Residual invariance	4731.39	1725	74.51	9	0.930	0.002	0.927	0.037	0.041 (0.040,0.042)
**Country**									
Configural invariance	7899.22	3296	–	–	0.905	–	0.895	0.044	0.052 (0.050,0.053)
Metric invariance	8150.93	3398	251.71	102	0.901	0.004	0.895	0.047	0.052 (0.050,0.053)
Scalar invariance	8656.23	3500	505.30	102	0.893	0.008	0.890	0.049	0.053 (0.052,0.055)
Residual invariance	8914.95	3527	261.72	27	0.888	0.005	0.886	0.062	0.054 (0.053,0.056)
**Skill level**									
Configural invariance	7261.64	3296	–	–	0.919	–	0.911	0.040	0.048 (0.047,0.050)
Metric invariance	7376.37	3398	114.73	102	0.918	0.001	0.913	0.042	0.047 (0.046,0.049)
Scalar invariance	7643.33	3500	266.96	102	0.915	0.003	0.912	0.043	0.048 (0.046,0.049)
Residual invariance	7707.94	3527	64.61	27	0.914	0.001	0.912	0.044	0.048 (0.046,0.049)

*Grouping variables were as follows: Gender: (1) male, (2) female; Country: (1) United Kingdom, (2) Australia, (3) United States, (4) Hong Kong; Skill level: (1) Beginner, (2) Amateur, (3) Semi-professional/County/State, (4) Professional/National/International.*

The data presented an appropriate fit to the SDCM-AA model; χ^2^(920) = 4472.22, CFI = 0.920, TLI = 0.914, SRMR = 0.047, RMSEA = 0.041 (90% CI = 0.040,0.042). In total, 54.0% of the variance in susceptibility to doping was explained in the model, and 44.8% of attitudes toward doping. Standardized parameter estimates accounted for contrasting amounts of this variance. Susceptibility to doping was positively predicted by attitudes toward doping (γ = 0.44, *p* < 0.001, 95% CI = 0.36,0.52) and by reference group (β = 0.44, *p* < 0.001, 95% CI = 0.37,0.51). Attitude toward doping was primarily predicted by cheating (β = 0.42, *p* < 0.001, 95% CI = 0.33, 50) and benefit (β = 0.25, *p* < 0.001, 95% CI = 0.18,31).

**FIGURE 1:**
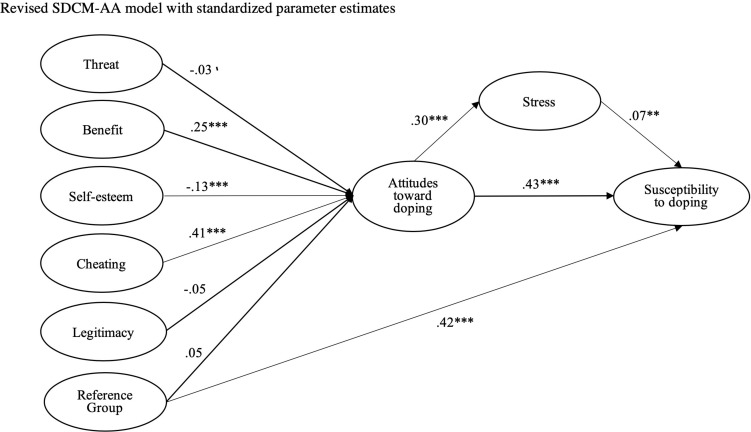
Revised SDCM-AA model with standardized parameter estimates.

Stress was identified as a significant predictor of doping susceptibility (Nicholls et al., 2019a). Perhaps then, rather than a moderator of doping attitudes, stress should be placed as a mediating variable between doping attitudes and doping susceptibility. Stress was entered for the revised SDCM-AA (see Figure 1), and although model fit was marginally improved, χ^2^(877) = 4033.11, CFI = 0.925, TLI = 0.919, SRMR = 0.055, RMSEA = 0.042 (90% CI = 0.040,0.043), variance explained in doping susceptibility remained the same (*R*^2^ = 0.54). Stress was only a small determinant of doping susceptibility (γ = 0.07, *p* < 0.01, 95% CI = 0.01,13), but it was significantly predicted by attitudes (γ = 0.30, *p* < 0.001, 95% CI = 0.23,36). The path from attitudes to susceptibility was largely unchanged (β = 0.43, *p* < 0.001, 95% CI = 0.36,51).

Multi-group SEMs for gender, country of residence, and skill level were examined to test structural invariance. That is, when the measurement model is constrained to be equal across groups, the structural paths in the model are freely estimated. Acceptable model fit indicates invariance across groups. Model fit for gender, χ^2^(1738) = 5254.07, CFI = 0.919, TLI = 0.916, SRMR = 0.058, RMSEA = 0.044 (90% CI = 0.043,0.045), and skill level, χ^2^(3544) = 8228.99, CFI = 0.904, TLI = 0.902, SRMR = 0.064, RMSEA = 0.050 (90% CI = 0.049,0.052), suggested only negligible group variance. There was, however, substantive group variance by country of residence, χ^2^(3544) = 9278.86, CFI = 0.881, TLI = 0.879, SRMR = 0.070, RMSEA = 0.056 (90% CI = 0.054,0.057). Standardized parameter estimates are presented in Table 2. Specifically, the United States sample was distinct in some structural paths from the other samples. Notably, the proportion of variance in susceptibility was higher in the United States sample (*R*^2^ = 0.71). The path from reference group to susceptibility was substantively higher [0.60 (95% CI = 0.42,0.78); rest = 0.35 to 0.46], as was the path from benefit to attitude [0.50 (95% CI = 0.31,0.70); rest = 0.16 to 0.34]. Also, this was the only sample in which the path from cheating to attitude was not statistically significant [0.08 (95% CI = −0.24,0.39); rest = 0.35 to 0.51].

**TABLE 2:** Multi-group SEM standardized parameter estimates (95% CI) for gender, country of residence, and skill level.

**Model**	**THR→ATT**	**BEN→ATT**	**EST→ATT**	**CHE→ATT**	**LEG→ATT**	**REF→ATT**	**ATT→STR**	**STR→SUS**	**REF→SUS**	**ATT→SUS**	***R*^2^ SUS**
**Gender**											
Male	−0.08 (−0.16,0.01)	0.25 (0.17,0.33)	−0.14 (−0.23,−0.06)	0.39 (0.28,0.50)	0.04 (0.12,0.05)	0.04 (−0.05,0.13)	0.28 (0.20,0.37)	0.10 (0.03,0.17)	0.41 (0.32,0.49)	0.46 (0.37,0.55)	0.58
Female	0.06 (−0.05,0.18)	0.26 (0.15,0.37)	−0.08 (−0.19,0.02)	0.45 (0.29,0.61)	−0.10 (−0.21,0.02)	0.07 (−0.05,0.18)	0.32 (0.22,0.42)	0.07 (−0.04,0.18)	0.42 (0.30,0.55)	0.36 (0.21,0.51)	0.46
**Country**											
United Kingdom	−0.03 (−0.13,0.06)	0.18 (0.10,0.27)	−0.10 (−0.19, −0.01)	0.42 (0.31,0.54)	−0.12 (−0.21,−0.04)	0.07 (−0.03,0.16)	0.25 (0.15,0.34)	0.09 (0.01,0.17)	0.38 (0.28,0.49)	0.46 (0.35,0.56)	0.53
Australia	0.09 (−0.04,0.22)	0.16 (0.03,0.28)	−0.12 (−0.32,0.07)	0.37 (0.06,0.67)	−0.04 (−0.19,0.11)	0.05 (−0.11,0.20)	0.30 (0.17,0.43)	0.19 (0.06,0.32)	0.46 (0.31,0.61)	0.30 (0.05,0.55)	0.46
United States	−0.18 (−0.32, −0.05)	0.50 (0.31,0.70)	−0.03 (−0.18,0.13)	0.08 (−0.24,0.39)	0.11 (−0.03,0.26)	0.16 (−0.09,0.40)	0.23 (0.03,0.44)	−0.06 (−0.23,0.11)	0.60 (0.42,0.78)	0.43 (0.23,0.63)	0.71
Hong Kong	−0.08 (−0.34,0.18)	0.34 (0.20,0.48)	−0.21 (−0.37, −0.06)	0.51 (0.33,0.69)	0.05 (−0.16,0.26)	−0.06 (−0.21,0.09)	0.42 (0.25,0.60)	0.06 (−0.12,0.24)	0.35 (0.17,0.53)	0.47 (0.26,0.68)	0.52
**Skill Level**											
Beginner	−0.25 (−0.51,0.01)	0.22 (−0.07,0.52)	0.02 (−0.30,0.34)	0.26 (−0.03,0.55)	0.03 (−0.23,0.29)	0.01 (−0.23,0.24)	0.17 (−0.06,0.40)	0.04 (−0.18,0.25)	0.26 (0.00,0.52)	0.49 (0.27,0.70)	0.37
Amateur	−0.01 (−0.10,0.08)	0.26 (0.19,0.34)	−0.13 (−0.21,−0.05)	0.43 (0.32,0.54)	−0.08 (−0.16,0.00)	0.04 (−0.05,0.13)	0.30 (−0.22,0.39)	0.11 (0.05,0.18)	0.42 (0.34,0.51)	0.42 (0.33,0.52)	0.55
National	−0.07 (−0.26,0.12)	0.20 (0.04,0.35)	−0.06 (−0.24,0.12)	0.31 (0.05,0.57)	−0.03 (−0.24,0.18)	0.15 (−0.02,0.32)	0.27 (0.14,0.41)	0.07 (−0.07,0.21)	0.50 (0.31,0.69)	0.38 (0.17,0.60)	0.58
International	−0.11 (−0.26,0.03)	0.33 (−0.17,0.49)	−0.22 (0.37, −0.06)	0.46 (0.23,0.69_	−0.05 (0.19,0.09)	0.05 (−0.12,0.23)	0.38 (0.17,0.60)	−0.05 (−0.25,0.15)	0.32 (0.10,0.54)	0.57 (0.32, 82)	0.57

*THR, Threat; BEN, Benefit; EST, Esteem; CHE, Cheating; LEG, Legitimacy; REF, Reference Group; ATT, Attitude; STR, Stress; SUS, Susceptibility. Statistical significance indicated by absence of zero within 95% confidence intervals.*

**FIGURE 2:**
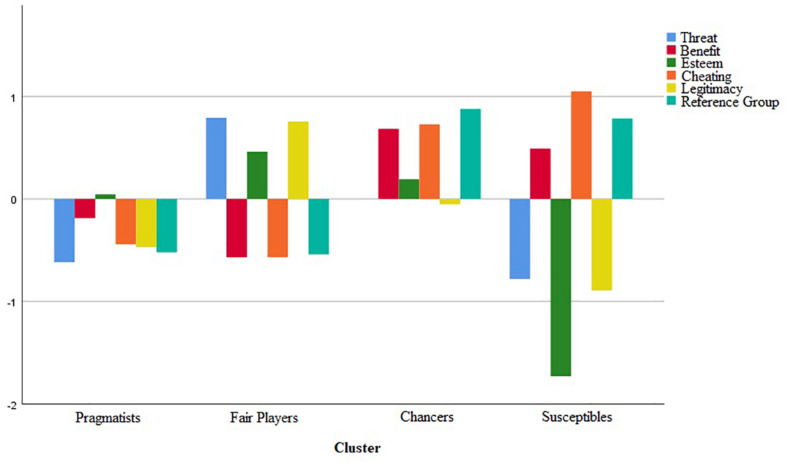
Sub-scale Scores for the Pragmatists, Fair Players, Chancers, and Susceptibles Clusters.

Subscale scores for the six predictors of attitudes toward doping were converted to *z* scores for cluster analysis. The dendrogram from hierarchical clustering presented a marked flattening, indicating the existence of four clusters. The subsequent non-hierarchical clustering technique presented the optimal four-cluster solution (see Figure 2). Participants gathered in Cluster 1 (*n* = 586) were distinct in that all of their *z* scores were average or low. These participants we relatively disengaged with doping overall. We labeled Cluster 1 as “Pragmatists”. Cluster 2 gathered participants (*n* = 726) who scored high on threat, esteem, and legitimacy, while scoring relatively low on benefit, cheating, and reference group. We named this cluster “Fair Players”. Participants gathered in Cluster 3 (*n* = 547) scored relatively high in benefit, cheating, and reference group, while having average *z* scores for threat, esteem, and legitimacy. We named this cluster “Chancers.” Finally, Cluster 4 gathered participants (*n* = 266) that, like the chancers, scored relatively high in benefit, cheating, and reference group, but, unlike the chancers, presented low *z* scores for threat, esteem, and legitimacy. We named this cluster “Susceptibles”.

**FIGURE 3:**
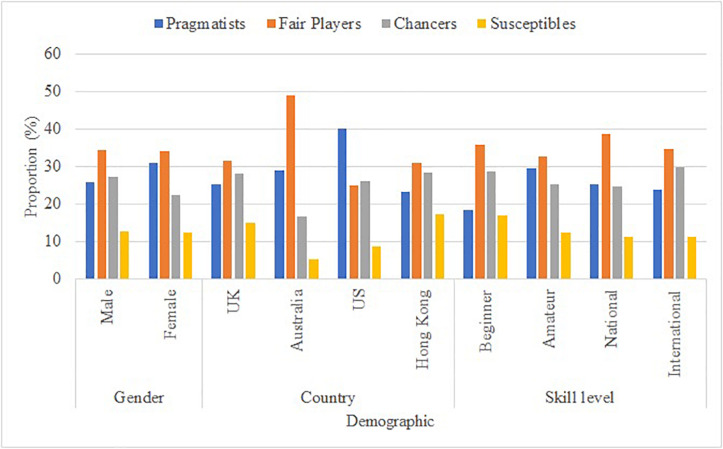
Proportion of Pragmatists, Fair Players, Chancers, and Susceptibles across Gender, Country of Residence, and Skill Level.

We next examined the demographic detail of each cluster to test distribution across gender, country of residence, and skill level using chi-square with 2000 bootstrapped samples. Distributions are presented in Figure 3. There was a small, negligible gender effect across clusters [χ^2^(3) = 9.21, *p* = 0.027, Cramer’s *V* = 0.066 (95% CI = 0.034,0.112)]. A larger effect was present for country of residence × cluster [χ^2^(9) = 13.85, *p* < 0.001, Cramer’s *V* = 0.128 (95% CI = 0.108,0.155)]. Notably, the Australian sample contain a much greater proportion of fair players relative to the other samples and the United States sample contained more pragmatists. A small, negligible effect was present for skill level × cluster [χ^2^(9) = 17.35, *p* = 0.044, Cramer’s *V* = 0.052 (95% CI = 0.042,0.086)].

A one-way ANOVA with 2000 bootstrapped samples measured differences between clusters on the variables of attitudes toward doping and susceptibility to doping. Significant differences were present between all clusters for both variables [attitudes: *F*(3,2100) = 188.20, *p* < 0.001, *n*^2^ = 0.21; susceptibility: *F*(3,2100) = 370.66, *p* < 0.001, *n* = 0.35]. All planned comparisons were statistically significant (*p* < 0.001, *p* < *q*) except for pragmatists vs. fair players for susceptibility. A summary of all comparisons is presented in Table 3. Overall, both attitude and susceptibility were vastly greater among athletes clustered in the Chancers and Susceptibles, than athletes in the Pragmatists and Fair Players cluster.

**TABLE 3:** Planned comparisons between clusters on attitudes and susceptibility toward doping.

	***M* (*SD*)**	**Pragmatists**	**Fair Players**	**Chancers**	**Susceptibles**
**Attitude**					
Pragmatists	6.37 (3.41)	–	0.32	0.63	1.21
Fair Players	5.42 (2.63	0.95	–	0.93	1.63
Chancers	9.20 (5.42)	−2.83	−3.78	–	0.45
Susceptibles	11.73 (6.12)	−5.36	−6.31	−2.53	–
**Susceptibility**					
Pragmatists	7.89 (4.27)	–	0.09	1.25	1.72
Fair Players	7.51 (4.14)	0.38	–	1.36	1.87
Chancers	15.27 (7.28)	−7.38	−7.76	–	0.30
Susceptibles	17.53 (7.78)	−9.64	−10.02	−2.26	–

*Mean difference is presented below the diagonal, and Cohen’s *d* is presented above the diagonal.*

Percentile scores are presented in Table 4. As moderating variables did not have a significant effect on doping factors, we did not calculate separate norms by demographic categories.

**TABLE 4:** Transformed (*t*) normative values for each ASDI scale.

**Percentile**	**Threat**	**Benefit**	**Esteem**	**Cheating**	**Legitimacy**	**Reference Group**	**Stress**	**Attitude**	**Susceptibility**
10	14	5	20	–	17	–	7	–	–
20	16	6	24	–	19	5	10	–	–
30	17	8	26	5	20	7	11	–	5
40	19	10	28	7	22	9	14	4	6
50	20	13	30	8	24	11	16	5	8
60	22	16	31	10	26	14	17	7	10
70	23	19	32	13	28	17	20	8	14
80	25	21	34	17	30	20	22	10	17
90	28	25	35	21	33	24	25	14	21

*Due to more than 10% accounting for the lowest possible score on some scales, not all scales are able to identify all percentiles.*

## Discussion

In this study, we examined the SDCM-AA (Nicholls et al., 2015), created psycho-social doping cluster scores, and generated norm values for adolescent athletes from the ASDI (Nicholls et al., 2019a). The data collected presented an appropriate fit for the revised SDCM-AA, as susceptibility toward doping was significantly and positively predicted by attitudes toward doping and reference group opinion. Attitudes toward doping were associated with cheating and benefit variables. Contrary to expectation, however, the moderator variables of participation level, gender, and stress had no real effect. In practical terms, this is quite beneficial, as we suggest that interventions designed to change attitudes do not necessarily need to be specific to such demographics. Country of residence did present as a moderating factor, perhaps a reflection on the sports played within each sample. Although the SDCM-AA predicted that stress was a factor that influenced doping attitudes, we did not find this. Rather, stress does not appear to influence attitudes toward doping, but is influenced by attitudes. We propose this as an alteration in the revised SDCM-AA.

Cluster analyses identified four distinct groups of athletes, which we termed the Susceptibles, Chancers, Pragmatists, and Fair Players. The Susceptibles are would-be dopers, as they have a cheating orientation and are prepared to identify with the benefits of doping. They are also highly influenced by their reference group, appraise little threat in doping, and have little faith in the legitimacy of drug testing. The Susceptibles are also characterized by low self-esteem, which may be a driver toward doping when combined with the other factors. The Chancers are also at risk of doping because they identified with the benefits of doping, scored high on willingness to cheat, and were highly influenced by their reference group. This group neither agreed nor disagreed that doping posed a threat in terms of their health or being caught and that testing procedures are legitimate. The Pragmatists refused to engage with any aspect of doping and were less likely to dope than the Susceptibles and Chancers, but more susceptible than the Fair Players. The Fair Players demonstrated high levels of sportspersonship, higher levels of self-esteem, and considered the system to be legitimate and represented a genuine threat to dopers. They also had little orientation toward cheating, saw little benefit of doping, and were less influenced by their reference group. This group was the least susceptible to doping. It is on this basis that we propose that recognition of these clusters can help inform anti-doping interventions. The contribution of all ASDI subscales to identifying clusters was significant and supports the retention of all subscales.

These clusters or athlete profiles could be useful to sporting organizations, national anti-doping organizations (NADOs), or education authorities in identifying athletes who have doped, currently doping, or who are at risk of taking PEDs in the future. We believe that the cluster grouping can be used to create individualized interventions, based on the athletes’ score on the different psycho-social variables. If, for example, certain athletes are deemed to have a profile that is related to being susceptible to doping or they have a favorable attitude toward doping, they could be exposed to an individualized education program, which reflects their scores on other elements of the SDCM-AA. Evidence from other domains, such as education and medicine, revealed that individualized interventions are superior to generic interventions (e.g., Qian et al., 2018; Chen et al., 2019; Partanen et al., 2019). Published interventions designed to reduce doping prevalence have not differentiated between individuals and thus considered individual athletes’ existing knowledge, attitudes, or susceptibility. Although the Athletes Training Learning and to Avoid Steroids (ATLAS; Goldberg et al., 1997) and Athletes Targeting Healthy Exercise and Nutrition Alternatives (ATHENA; Elliot et al., 2008) were gender-specific interventions, the content for ATLAS and ATHENA was standardized. The ATHENA program was effective in reducing substance use 1–3 years after graduating high school, but the effect sizes were small (Ntoumanis et al., 2014). In addition to doping interventions being individualized, Hallward and Duncan (2018) suggested that they should be collaborative, start early, and be both engaging and interactive. The development of educational programs is crucial to help reduce doping behaviors via reducing attitudes and susceptibility toward doping.

We also generated norm values, created by the ASDI. This represents a way of identifying athletes who might be at risk of committing doping offenses. Scores produced by the ASDI could then be used to benchmark athletes. For example, a score greater than 14 for susceptibility on the ASDI means an adolescent athlete is more susceptible than 70% of his or her peers. Alternatively, a score of 25 or more for benefit means that an athlete identifies with the benefits of doping more than 90% of his or her peers. Understanding what constitutes a high score in each factor of the SDCM-AA is important to predict at-risk athletes. Until now, this information is currently unavailable for NADOs, sporting organizations, or coaches, but has the potential to shape education by making it athlete specific, as opposed to being generic.

A strength of this current research relates to the participant and aligns to calls made by Rad et al. (2018) for making psychological research more representative of the human population, which generally relies on the Western population and featuring participants from just one country. This is also evident within the doping literature, where samples generally consist of athletes from the same country (Nicholls et al., 2017a). This does not allow scholars to identify differences across countries, which is important in terms of developing appropriate interventions. We found evidence of differences among country of residence. In particular, the Australian sample contained a higher proportion of Fair Players in comparison to the other countries, whereas the United States sample contained more Pragmatists, in comparison to the other countries. This may be due to differences in the sports played among our sample, but further research is required to examine this further and to identify possible reasons.

## Limitations

A limitation of this study relates to potential sample bias, as 292 athletes who received a questionnaire chose not to participate. It is unknown why these athletes chose not to participate in this research, and this could raise issues regarding the validity of the data. The response rate of 88% compares favorably to other studies examining the psycho-social factors associated with doping, such as Giraldi et al. (2015) who reported a response rate of 76.91%, but inferior to other research with response rates of 100% (Blank et al., 2016) and 95% (Mudrak et al., 2018). Although the sample contained more athletes from the United Kingdom, unlike many studies within the doping literature, our study includes athletes from multiple countries and across four continents. This aligns with Rad et al. (2018), who recommended making psychological science more representative of the human population.

Another limitation relates to the reliance on cross-sectional, self-reported data on the psycho-social variables associated with doping. This yields two potential limitations: common method bias and social desirability. By common method bias, we refer to the extent the model may be a reflection on the measurement of the constructs rather than the constructs themselves. It is very challenging to test against an objective, observable criterion in doping research. We must therefore remain conscious of this issue. In order to limit the effects of social desirability, all questionnaires were completed anonymously, and participants did not report their name. Indeed, scholars such as Ntoumanis et al. (2017) have argued that self-reports are the most realistic way of assessing constructs in psychological research. Notwithstanding this, a limitation of this study relates to the lack of information around doping prevalence, which we did not assess. As such, it would be useful to identify the constructs within the revised SDCM-AA (Nicholls et al., 2015) that predict doping prevalence and whether the Susceptibles are more likely to dope than the Pragmatists or the Fair Players. It should be noted, however, that scholars (e.g., Jackson, 1998; Andrews et al., 2008; Cranford et al., 2010; Jackson et al., 2016) have suggested that substance use can be indirectly inferred by proxy measures such as susceptibility among adolescents. Further research is required to assess this among adolescent athletes.

A possible limitation of the SDCM-AA and the SDCM (Donovan et al., 2002) is that both models include personality as a factor that is associated with doping attitudes, with self-esteem being the key personality factor that predicts doping attitudes. Other scholarly activity has revealed that other personality factors are associated with doping attitudes such as perfectionism (Madigan et al., 2016), risk-taking propensity (Jalleh et al., 2014), and honesty and humility (Nicholls et al., 2019a). Further, doping attitudes have also been associated with a taxonomy of personality traits, the Dark triads (Nicholls et al., 2017c, 2019b). It appears that personality may play an important role in shaping attitudes toward doping, so the SDCM and the SDCM-AA may need revising as other research identifies personality factors and alternative taxonomies of personality traits that are associated with doping attitudes and doping susceptibility.

## Conclusion

The revised SDCM-AA appears as a suitable model that helps explain the factors associated with doping attitudes and doping susceptibility. It is also one of the first doping models that includes stress. We identified four different clusters of athletes (e.g., Susceptibles, Chancers, Pragmatists, and Fair Players), which quantifies the proportion of athletes who are at high, relatively high, medium, and low risk of taking PEDs. NADOs, sports federations, and coaches could use the ASDI (Nicholls et al., 2019a) to identify the Susceptibles and Chancers, and expose these athletes to anti-doping education interventions. Hopefully, this education would take place before they have engaged in doping practices. Furthermore, anti-doping interventions could be developed based on the four clusters, so they are targeted for the athlete. Finally, we created norm values for the sub-components of the SDCM-AA. These values can be used as a benchmark for organizations or individuals such as coaches who want to make comparisons between their athlete’s score with a larger sample.

## Data Availability Statement

The datasets available on request from the corresponding author.

## Ethics Statement

The studies involving human participants were reviewed and approved by Department of Sport, Health and Exercise Science Ethics Committee. Written informed consent to participate in this study was provided by the participants’ legal guardian/next of kin.

## Author Contributions

All authors assisted with data collection. AN conceptualized and contributed to the writing. JP conceptualized, analyzed, and contributed to the writing. AL, RM, CS, LJ, TB, and MT contributed to the writing.

## Conflict of Interest

The authors declare that the research was conducted in the absence of any commercial or financial relationships that could be construed as a potential conflict of interest. The handling editor declared a past co-authorship with one of the authors AN.

## Footnotes

1 The SDCM-AA (Nicholls et al., 2015) included a *morality* construct. In the development of the ASDI, this gave way to a scale we termed as *cheating*, as items were phrased so a high score represented an orientation toward cheating more than they represented morally virtuous reasoning or behavior.
